# Scheimpflug topographical changes after Femtosecond LASIK for mixed astigmatism – theoretical aspects and case study

**DOI:** 10.22336/rjo.2017.13

**Published:** 2017

**Authors:** Bogdana Tabacaru, Horia Tudor Stanca

**Affiliations:** * ”Prof. Dr. Agrippa Ionescu” Emergency Hospital, Bucharest, Romania; ** “Carol Davila” University of Medicine and Pharmacy, Bucharest, Romania; *** ”Metropolitan” Hospital, Bucharest, Romania

**Keywords:** Femtosecond-LASIK, Mixed Astigmatism, Scheimpflug Analysis, Corneal Topography, Tangential Anterior Map

## Abstract

**Objective:** To evaluate the corneal topographical changes after Femtosecond-LASIK surgery in eyes with mixed astigmatism.

**Methods:** We present the analysis of the corneal Scheimpflug topographies of a patient treated with Femtosecond-LASIK technique for bilateral mixed astigmatism.

**Results:** Three-dimensional reconstruction maps and differential anterior curvature maps were used to demonstrate the ablation profile and its stability in time.

**Conclusions:** Visual and refractive results were very good after surgery, being topographically confirmed by the corneal reshaping which was performed as planned, the achieved ablation being stable during the one-year follow-up period.

## Introduction

Astigmatism is the condition of refraction in which the rays of light coming from a point source cannot produce a point on the retina [**1**,**2**]. Cornea is the major source of astigmatism in the optical system as it is responsible for about 74% of the total dioptric power of a normal eye [**3**]. The optical power of the mixed astigmatic eye is different in two principal meridians, perpendicular to one other, one meridian being myopic and the other being hyperopic [**4**]. The aim of corneal refractive surgery in mixed astigmatism is to reshape the cornea, flattening it in the myopic meridian and steepening it in the hyperopic meridian [**5**].

After the corneal refractive surgery, changes in the corneal shape and curvature can be evaluated by using a variety of devices based on Placido-disc systems and elevation analyzers [**6**]. The Schwind Sirius® (Schwind Eye-Tech-Solutions GmbH&Co, Germany) is a device that combines a Scheimpflug camera with a Placido disc corneal topographer [**7**]. Placido-based videokeratoscopy measures the corneal reflection of mires (circles of light) of known radius, the corneal power being estimated mathematically. Rotating Scheimpflug camera system uses the slit illumination to obtain an optical section that is captured in a side view, the camera being oriented according to the Scheimpflug principle, in order to create sharp images from anterior corneal surface to depth [**8**]. Data obtained after corneal scanning are converted to computerized color scale maps [**6**].

The axial (or sagittal) map is the most commonly used map for routine screening, as it easily classifies the normal and abnormal corneas and differentiates between spherical, astigmatic or irregular corneas. Due to the Placido rings configuration and to the axial acquisition which intersects with the instrument axis, the sagittal map fails to describe the true shape and power of the peripheral cornea [**6**,**9**].

The tangential map (also called “instantaneous radius of curvature”) has a better accuracy in the evaluation of the peripheral changes in shape and curvature but has the tendency to reveal excessive details that are not always clinically relevant. It may be very useful in detecting mild corneal changes that could not be detected by the sagittal maps [**6**,**9**].

**Fig. 1:**
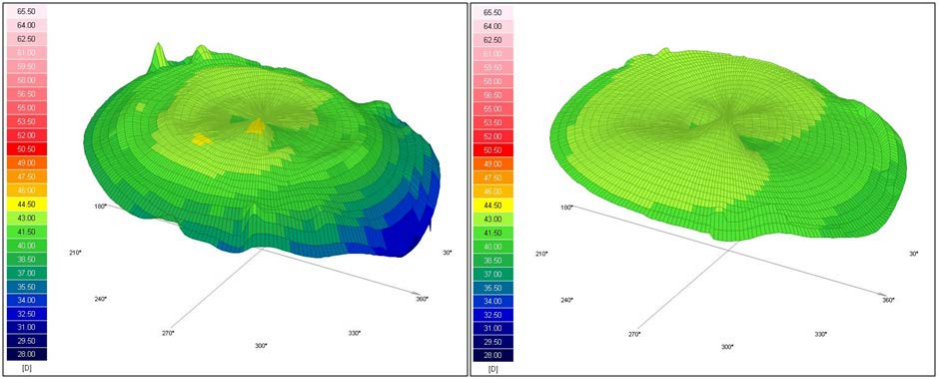
Scheimpflug topography of a spherical cornea. Left image – tangential anterior map; Right image – sagittal anterior map

The three-dimensional reconstruction maps, available in both sagittal and tangential acquisitions, offer an overall view and a better understanding of the real corneal shape with steep and flat areas [**9**]. Normal corneas are prolate, being steeper centrally and flatter peripherally, with a medium anterior refractive power of 43.00-43.50 diopters [**10**]. **Fig. 1** shows the three-dimensional tangential anterior and sagittal anterior configuration of a normal spherical cornea.

## Material and methods – Case study

A 23-year-old white woman (P.A.M.), underwent bilateral Femtosecond-LASIK with the VisuMax® – Mel80® platform (Carl Zeiss Meditec, Germany), to correct mixed astigmatism. Preoperative manifest refractive errors were +1.75 – 3.25 x 0o in the right eye and +1.75 – 3.75 x 175o in the left eye. The best-corrected visual acuity of both eyes was 20/20. The patient underwent refraction with fogging and refraction under cycloplegia, which demonstrated a hyperopic shift of 0.5 and 1.25, respectively in both eyes. Keratometry values for the right eye were K flat 41.23 x 8o, K steep 44.28 x 98o and for the left eye K flat 41.20 x 177o, K steep 45.05 x 87o. Corneal pachymetry in the thinnest location was 0.565 mm in the right eye and 0.561 mm in the left eye. Topographies were performed with the Schwind Sirius® topographer. Preoperative slit-lamp biomicroscopy and mydriatic fundoscopy revealed no pathological findings. 

Femtosecond laser parameters for the anterior corneal flap cutting were chosen as it follows: depth of 120 μm, diameter of 8.8 mm, hinge of 3.84 located superiorly and side cut angulation of 50o. We have chosen a refraction of +2.25 – 3.25 x 0o in the right eye and +2.25 – 3.75 x 175o in the left eye for the excimer treatment plan, with an optical zone of 6.5 mm for both eyes. Surgery was uneventful.

Postoperative examinations were carried out on the first day following the surgery and then after one, six and twelve months. We evaluated the uncorrected distance visual acuity (UDVA), the manifest refraction and we performed slit-lamp examinations at each visit. Except for the first postoperative day, topographies were achieved at each follow-up visit.

## Results

Postoperative results were good, with full recovery of uncorrected vision and a manifest refraction very close to emmetropia. The keratometry was constant over the follow-up period (**Table 1**).

**Table 1:** Postoperative visual acuities, manifest refraction and keratometric measurements for patient P.A.M.

Postoperative visit	UDVA	Manifest refraction	K flat	K steep
Right eye:				
- 1 day	20/ 20	+0.75 –1.00 x 87o	42.50 x 97o	43.25 x 7o
- 1 month	20/ 20	+0.50 –0.25 x 44o	42.75 x 90o	43.00 x 180o
- 6 months	20/ 20	+0.75 –0.25 x 33o	42.75 x 0o	42.75 x 90o
- 12 months	20/ 20	+0.75 –0.25 x 19o	42.50 x 110o	43.00 x 20o
Left eye:				
- 1 day	20/ 20	+0.25 –0.75 x 173o	42.75 x 179o	43.00 x 89o
- 1 month	20/ 20	+0.25 –0.50 x 168o	42.50 x 163o	43.25 x 73o
- 6 months	20/ 20	+0.75 –0.25 x 177o	42.75 x 168o	43.25 x 78o
- 12 months	20/ 20	+0.50 –0.50 x 176o	42.75 x 167o	43.25 x 77o

We further present the corneal topographies performed pre and postoperatively and their analysis regarding the corneal shape and curvature changes occured after the refractive surgery and their stability over the follow-up period.

**Fig. 2:**
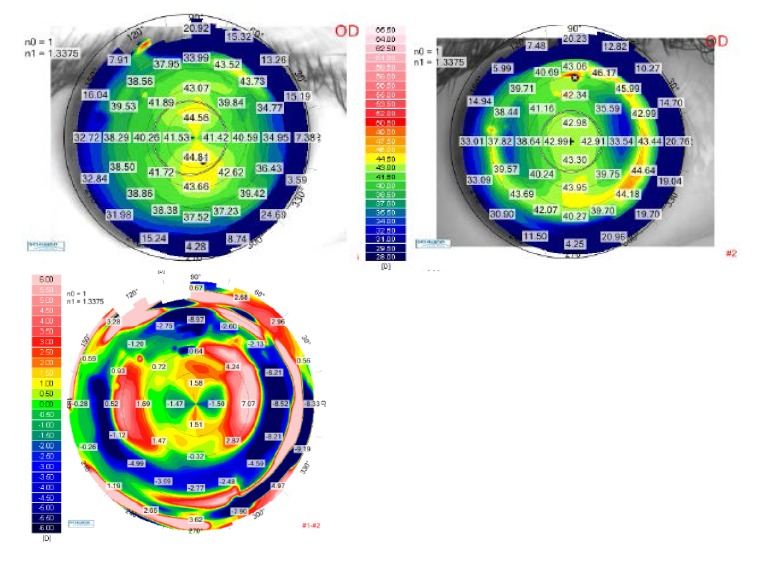
Up-left: Tangential anterior map for the right eye of the patient P.A.M. at the preoperative visit. Up right: Tangential anterior map for the right eye of the patient P.A.M. at 1-month postoperative visit. Bottom: Differential tangential anterior map between preoperative and postoperative 1 month visits, for the right eye of the patient P.A.M.

**Fig. 3:**
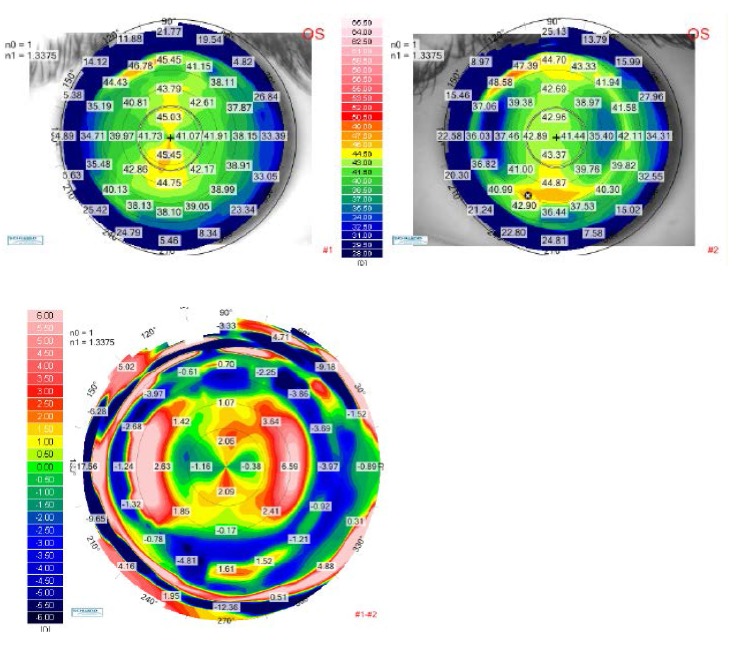
Up-left: Tangential anterior map for the left eye of the patient P.A.M. at the preoperative visit. Up right: Tangential anterior map for the left eye of the patient P.A.M. at 1-month postoperative visit. Bottom: Differential tangential anterior map between preoperative and postoperative 1 month visits, for the left eye of the patient P.A.M.

**Fig. 4:**
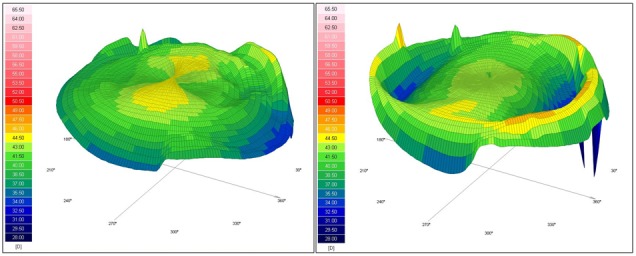
Tangential anterior map of Scheimpflug topography of the right eye of patient P.A.M. Left image – Preoperative; Right image – one-month postoperative

**Fig. 5:**
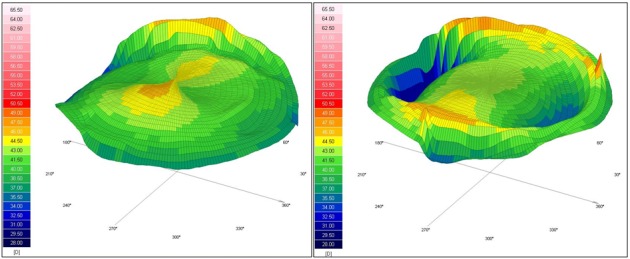
Tangential anterior map of Scheimpflug topography of the left eye of patient P.A.M. Left image – Preoperative; Right image – one-month postoperative

**Fig. 2** and **3** demonstrate the results of the calculated ablation we have performed for the right eye and the left eye respectively. The preoperative tangential anterior map was on the upper-left and the one-month postoperative tangential map was on the upper-right. As the color scale was the same for both topographical aquisitions, we were able to directly compare the two scans on a differential map, visualizing the ablation profile. The relative shape of the corneas was changed with flattened areas in the myopic meridians and steepened areas in the hyperopic meridians. As a result of the surgery, the corneas theoretically looked as if they were spheres. The corneal shape changing result can be better understood in the three-dimensional reconstruction, shown in **Fig. 4** for the right eye and in **Fig. 5** for the left eye. 

**Fig. 6:**
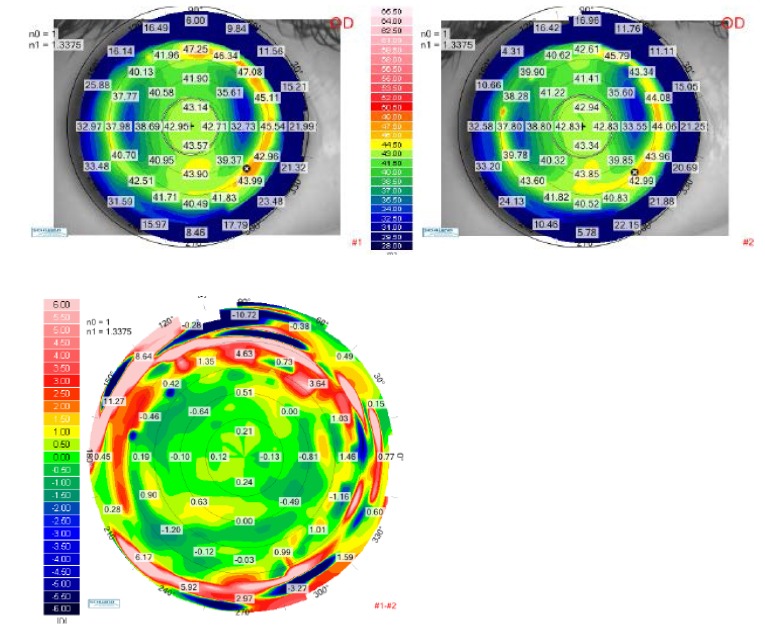
Up-left: Tangential anterior map for the right eye of the patient P.A.M. at 1-month postoperative visit. Up right: Tangential anterior map for the right eye of the patient P.A.M. at 12 months postoperative visit. Bottom: Differential tangential anterior map between postoperative 1 month and 12 months visits, for the right eye of the patient P.A.M.

**Fig. 7:**
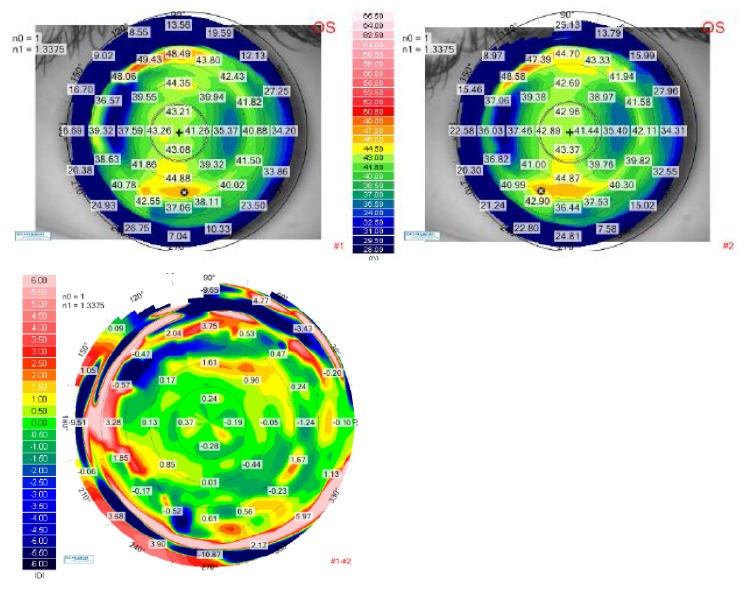
Up-left: Tangential anterior map for the left eye of the patient P.A.M. at 1-month postoperative visit. Up right: Tangential anterior map for the left eye of the patient P.A.M. at 12 months postoperative visit. Bottom: Differential tangential anterior map between postoperative 1 month and 12 months visits, for the left eye of the patient P.A.M.

Two postoperative corneal topographical maps were also compared in order to demonstrate the ablation stability in time. The postoperative differential maps for both eyes were achieved by substracting the 12-months postoperative map from the one obtained at the 1-month follow-up visit. The postoperative differential maps displayed on the bottom of **Fig. 6** (for the right eye) and **Fig. 7** (for the left eye) show exactly the changes that had occured in every corneal point during the 1-year follow-up. The ablation profile was stable, with unsignificant changes of the radius of curvature inside the optical zone. 

## Discussion

Nowadays, corneal topography is an indispensable investigation in the preoperative and postoperative refractive surgery management. The curvature maps, available in bi- and three-dimensional imaging are very useful to better understand the corneal shape changes that occur after surgery. Modern software allows a comparative analysis of the corneal corresponding points and generates differential maps that are useful in the precise evaluation of changes between preoperative and postoperative visits or between two postoperative moments.

In the presented case, Femtosecond-LASIK technique was a suitable option for the correction of mixed astigmatism. During the entire follow-up period of one year, the uncorrected vision was 20/20, the manifest refraction was very close to emmetropia and the keratometry had constant values. The topographical maps used in the analysis demonstrated a proper ablation profile and its stability within at least one year.

**Disclosures**

The authors have no financial or proprietary interest in any device presented in this study.
